# Role Performance of Community Health Volunteers and Its Associated Factors in Kuching District, Sarawak

**DOI:** 10.1155/2017/9610928

**Published:** 2017-02-13

**Authors:** Melvin Hsien Liang Chung, Helmy Hazmi, Whye Lian Cheah

**Affiliations:** Department of Community Medicine and Public Health, Faculty of Medicine and Health Sciences, Universiti Malaysia Sarawak, 94300 Kota Samarahan, Sarawak, Malaysia

## Abstract

The objective of this study was to assess the role performance among KOSPEN community health volunteer in Kuching district and its associated factors. This was a cross-sectional study, conducted in 21 localities in Kuching with a total of 210 respondents. Data were collected using validated interviewer-administered questionnaires and analyzed using SPSS version 22.0. The respondents comprised 55.2% females, 81.9% married, and 41.4% aged above 45 and above and 72.4% completed their education up to secondary school. The result revealed that 59.0% of the respondents agreed and understood their role performances. Multiple Logistics analysis revealed that factors associated with role performance were age group (*p* = 0.003), education level (*p* < 0.001), marital status (*p* = 0.025), prestige and respect (*p* = 0.012), being seen as “doctor” in community (*p* = 0.003), job aids (*p* = 0.009), training location (*p* = 0.001), and supervision by community (*p* < 0.001). To increase and maintain the work performance of CHVs, commitment from the government, policy makers, stakeholders, and the communities is required.

## 1. Introduction

The concept of community-based health volunteer system has gained its popularity in developing countries to overcome the increasing demand for health care services and the shortage of formal health care providers [1]. In line with the Alma Ata declaration of Primary Health Care Concept (PHC), all community health strategies designed must address community needs at the local level and be led by the community members themselves, with the belief that health problems cannot be solved by distance policymakers and health planners but require the involvement of communities to mobilize local resources for optimal health [2].

WHO had demonstrated the important role of CHVs in achieving goals related to health indicators in Millennium Development Goals (MDG), for example, in MDG 4 to reduce child mortality, MDG 5 to improve maternal health, and MDG 6 to combat HIV/AIDS, malaria, and other diseases. Research has shown that CHVs tend to be actively involved in communicable disease control programmes such as HIV awareness campaign, dengue control, malaria, dehydration resulting from diarrhoea, and also increased immunization coverage [3, 4]. In low- and middle-income countries, CHVs have been found to be effective in reducing neonatal mortality [5] child mortality attributable to pneumonia and mortality caused by malaria [6, 7]. Meanwhile, in eastern Uganda, CHV had performed reasonably well in vertical programmes targeting single diseases, mainly for treatment of malaria [8, 9] and in pilot studies for treatment of pneumonia of childhood illness [10, 11]. Similarly in South Africa, CHVs are actively involved in the programmes that target infectious conditions such as HIV/AIDS and Tuberculosis (TB) as well as maternal and child health. According to Singh, their roles in such programmes are clearly defined and are established based on evidence illustrating the benefits and success [12].

In Malaysia, Ministry of Health has recently launched a health programme named KOSPEN (Komuniti Sihat, Perkasa Negara) which means a healthy community is the strength of a country, in 2013. This programme has the objective to transform public health services through an aggressive approach to the establishment of functional units consisting of volunteers from communities across the country that will serve as a health agent of change. The main strategies for this programme are to improve awareness among individual and community on noncommunicable diseases (NCD) and their risk factors, basic preventive measures, and detection of risk; transformation of health knowledge to healthy behaviour and lifestyle; and creating healthy lifestyle environment. KOSPEN consists of 5 main components which are eating healthy, active lifestyle, no smoking, weight management, and NCD detection through health screening. The volunteers are from their own village, and they are not paid or given any incentives or allowances. The whole concept is voluntary basis. They are preferred to be 15 years old and above, able to give full commitment as a health volunteer, able to read and write, and permanent residents of the village and there are no personal, political, and religion interests. They will undergo a course of health training and will be provided with job aid tools such as blood glucose monitoring kits, weighing scales, and measuring tapes for the routine health screening. Since 2013, there are a total of 45 villages proposed in the health programme KOSPEN in Kuching division; however, to date, only 21 villages had launched the programme. Each of the localities consists of ten community health volunteers (CHVs).

The existing literature on CHVs' performance is scanty. Globally, standard governmental monitoring and evaluation is based on monthly reporting rate, health knowledge, and percentage of household coverage which were found to focus too much on the job per se, leading to the omission of other important components of overall performance [13]. As such, Milkovich and Boudreau [14] recommended the use of “roles” to replace “job.” According to the role theory, individuals' role expectations are influenced by both their personal attributes and the context in which they exist [15].

Like any community-based programme, the success of the programme depends on the factors influencing the role performance of CHVs themselves. Factors such as sociodemographic status, motivation (extrinsic and intrinsic motivator, altruism, skill, and performances), and organizational input (selection and recruitment process, training, and supervision) play an important role in making sure CHVs perform in contributing to the overall performance of the organization [16–22]. Besides, all these factors, knowledge is another important component that associated with the role performance. Generally, CHVs with good knowledge tend to have good role performance. For example, in the disaster preparedness plan, it has become an essential issue for everyone to be involved since the incidence of natural disasters such as flood, draught, and earthquakes is becoming increasingly common. Therefore, CHVs have become the front liners to help the community in the emergency phase to prevent loss of life [23]. However, DeSimore reviewed that the assistance from the CHVs during a disaster event was restricted due to their limited knowledge and skills [24]. Hence, CHVs should be prepared and have the basic knowledge required to think critically and respond accordingly [25]. As a conclusion, knowledge gained is important in influencing the role performance of the CHV. Failure to address any of these factors can result in poor performance, high drop-out rate, and poor submission of programme report which eventually disrupt the continuity of the programme [19, 26, 27].

KOSPEN has been implemented for two years. With the aim to recruit 50,000 health volunteers by the year 2016, it is timely to access the role performance of our CHVs and determining its associated factors. The objective of this study is to measure the role performance of KOSPEN community health volunteer and its associated factors in Kuching district.

## 2. Materials and Methods

It was a cross-sectional study conducted in urban region of Kuching district from January until June 2016. Kuching is the capital and the most populous city of Sarawak, Malaysia. It is located on the Island of Borneo with a population of about 325,132. It has a diverse ethnicity composition of more than 40 ethnic groups, with Iban, Chinese, Malay, Bidayuh, Orang Ulu, and Melanau as major ethnic groups. In this district, there is one divisional health office, known as the Kuching Divisional Health office and few types of health services in the city, such as the main public hospitals, public health clinics, mobile clinic, flying doctor service, village clinics, and 1Malaysia clinic. The main hospital is the Sarawak General Hospital which is the oldest hospital since 1923. Another hospital is Rajah Charles Brooke Memorial Hospital and Hospital Sentosa. There are four private hospitals. Normah Medical Specialist Centre in Petra Jaya is the largest private hospital with 130 beds in Sarawak. In addition, three other large private health facilities are Borneo Medical Centre with 120 beds, Timberland Medical Centre with 100 beds, and KPJ Healthcare with 75 beds. The Kuching Divisional Health Office are responsible for managing the Community Health Volunteer who are responsible for each catchment area.

The target population of this study was the community health volunteer under KOSPEN programme in Kuching district. The sampling frame consisted of 21 villages with 10 CHVs each. Only those who were trained by respective District Health Office and registered with the Kuching Divisional Health Office were recruited in this study. No sampling was done. All the 210 CHVs were invited to participate. Prior to the data collection, the researchers approached the village headman for permission to enter and conduct the study in the village. The objectives of the study were brief and explained to all respondents. Data was collected face-to-face interview using questionnaire adopted and adapted from Chatio [27] and Adisa [28]. The questionnaire consists of five sections: sociodemographic profile (7 questions); motivation (5 questions); organization input (9 questions); knowledge (10 questions); and role performance (7 questions). For the knowledge component, summation of the questions answered correctly will be the score for the CHVs' knowledge on the program. Likewise, summation of the score for each of the questions on role performance will determine the level of their performance. All the data were analyzed to see the frequency and relative frequency for each factor accordingly and to see its correlation with role performance. A pretest of the questionnaire was carried out in a sample of 30 from another district. The feedback received indicated the questions were easily understood with no ambiguities. A minor modification was done to improve the phrasing of some questions.

Data analysis was performed using Statistical Package for Social Sciences (SPSS) version 22.0. All data was cleaned and checked for normality prior to analysis. Descriptive and inferential statistics were carried out with *p* < 0.05 significance. From the result, in view of the role performance scores being skewed, therefore, it was categorized into 2 levels of high and low performance using its median as cut-off point. Volunteer whose role performance score is above the median will be categorized as high performance and vice versa.

Ethical approval was obtained from Medical and Ethics Research Committee of Universiti Malaysia Sarawak. All respondents were brief and consent was obtained. Any information obtained from this study will remain confidential and will not be disclosed.

## 3. Results

A total of 210 respondents participated in this study with mean age of 42.6 years (SD 12.85). Majority of them were 35 years and above (72.4%), married (81.9%), have education level up to secondary school and above 88.1%, and worked as health volunteer more than 3 months (95.2%). Further details of sociodemographic profile can be found in Table 1.

Table 2 shows the profile regarding the motivation and attrition factors, knowledge, and organization input among the respondents answering “Yes” as their answer. Among the motivating factors, the top three factors that have the highest rating were* to improve the health of others* (97.1%),* help members/sick people* (96.7%), and* gain knowledge and practice skills learned* (90%). Under attrition factors,* no time* (72.4%),* transportation issue* (65.2%), and* job aids* (54.3%) were the top three rated. For factor such as knowledge, majority of the respondents answered correctly (79–99%) for all questions except for* regular physical exercise causes you suffering from stroke, DM and hypertension* (58.6%). About 40% of the respondents were selected by village heads. More than 70% of the respondents reported that they had adequate training, usually 1-2 days of duration. However, 53.3% of them found the training is acceptable. More than 60% of them were supervised by community members or village heads and are visited by their supervisor once a month (70.5%). About 83–85% of them felt supervision had helped them to be more motivated and committed to the programme.

Majority of the respondents were positive about their role performance with rating of each item under assessment of role performance more than 89% except for administrative works (53.3%). The total score for role performance was categorized into 2 levels of high and low performance using its median as cut-off point. Volunteer whose role performance score is above the median will be categorized as high performance and vice versa. Majority of them (59%) agreed and understood their role and responsibilities in the programme (refer to Table 3).

Multivariate logistic analysis was carried out using role performance (high and low) as dependent variables and selected independent variables screened by univariate analysis. The findings showed that only age group (*p* = 0.003), education level (*p* < 0.001), and marital status (*p* = 0.025) were the main factors associated with the role performance (Table 4). It is shown that respondents at age group of 35–44 years are 4.93 times higher performance compared to age group 45 and above (95% CI = 1.784, 13.660). On the other hand, group below 24 years old are 27.3 times higher performance as compared to the group of 45 years and above (95% CI = 2.781, 267.945). Majority of the respondents with tertiary education background have higher performance for 4.292 times compared with those with primary education (95% CI = 1.038, 17.751). Regarding their marital status, the result also highlighted that those who are married have 4.656 times higher performance as compared to those who are single (95% CI = 1.006, 17.588).

For extrinsic motivation factors, the data showed that prestige and respect factors motivated the respondents to perform 2.76 times better than those who were not (95% CI = 1.244, 6.120). Recognition as “doctor” in the community also motivated them to continue performing better as high as 8.65 times as compared to those who do not agree to this (95% CI = 2.044, 36.577). In addition, sufficient supplies of job aids such as boots, raincoat, and medical equipment influenced the respondents at 8.65 times higher performance as compared to those who do not think job aids can influence their work (95% CI = 1.324, 7.008). The respondents also revealed that the training location is important to affect their services especially those trainings carried out in community setting which have 4.76 times higher performance than those in government setting (95% CI = 1.930, 11.725). In this study, the community members/leaders played a more effective role than medically trained personnels in field supervision of CHV (OR = 7.15, 95% CI = 2.832, 18.055).

## 4. Discussion

This study revealed that CHVs' role in the community can affect CHV performance. This finding is consistent with other studies, indicating lack of understanding of role responsibilities which lead to poor performance and high attrition rate [29, 30]. It is through their understanding and commitment that high performance can be achieved.

The age groups with the best role performance are ranging from 35 to 44 years and 24 years and below. Age has been found in few literatures that older volunteers tend to be more motivated principally by altruistic motives, otherwise known as the values function [31], while young volunteers appear to be principally more easily influenced and encouraged by their peers or spouse and are more driven by career, social, and understanding values [32]. Finkelstein and colleagues also found that these career functions had higher motivating impact compared to the value function [31]. Besides, value function such as altruistic value is varied according to individual, while career function is a target or an objective that drove the young volunteer to work harder and perform better.

This study also reported that respondents with higher education are more likely to perform better than those with primary or secondary education (*p* = 0.022). In other words, higher education background is a positive factor that makes CHVs able to do the work better and improve their performance. A high level of education will help them to understand more in health knowledge, which is one of the performance indicators in this study. In addition, CHVs with a higher educational status would easily understand how to write and submit their monthly report. Kok et al. (2014) also agreed on the importance of CHVs' educational status, including literacy level, in maintaining their high performance. Therefore, a certain level of education should be one of the criteria in the selection of CHVs.

Based on the result, it is shown that marital status is another factor that influences the role performance. It was found in other studies that CHVs who are married have better support in managing household duties compared to those who are not married, which explained why the drop-out rate was lower [16, 19, 33]. However, there was also study that indicated otherwise where no difference was found between married and unmarried CHVs [34]. Perhaps these differences could have been due to cultural perception on the role of married person in work force.

This study revealed that recognition as “doctors” at the community level and the prestige and respect given by community members motivate them to perform better [OR = 2.76 (95% CI 1.244, 6.120)]. These findings were supported by Rowe et al. (2005), whete factors that motivate and directly influence retention and attrition rates of health volunteers are respect gained not only from health workers but also from the community [35]. On the other hand, Rahman et al. (2010) also found similar conclusion that recognition and respect at the community level and the status attached to being a health volunteer motivates the volunteers into community health interventions [19].

Lack of means of transport and job aids are factors that can influence CHVs' motivation and subsequently affect their performance. The cost of travel and replenishment of the supplies, material, and equipment are important determinants of their performance that should be taken into consideration. For example, in Malawi, lack of transport prevented some of the CHVs from covering some of the villages in their catchment areas and from obtaining drugs and other needed supplies from their respective health centres [36]. When any of the resources or supply of needed materials is disrupted, it will not only decrease their productivity but also lead to consequences such as losing the respect of the community without which a CHV can rarely be productive. In other words, CHVs need the trust of the community. When this is compromised CHVs become ineffective.

The result reported that having training at the community setting had more impact on the learning process and motivation for better understanding on the current issue in the community and subsequently intervene. This can increase their performance for 4.76 times better as compared to those trained at government setting (95% CI = 1.930, 11.725). Training centres in a local community setting are better than training centres located in a government facility, which may be logistically challenging for rural CHVs. The preference for a local training centres may reflect the logistical challenges faced by CHVs in the Kuching division, despite being a state capital city and the financial burden of CHVs as a result of having to travel. Most of the respondents do not have any fixed income.

The result also highlighted that supervision is an important factor to demonstrate better performance. Yet supervision is often neglected in a CHV programme. Ineffective supervision contributes to low CHV morale and poor productivity. For example, supervision of CHVs in the Republic of Zambia's Kalabo District did not have a positive impact on performance because the quality was poor and almost half the CHVs did not experience any benefit from the supervision visits [37]. The study also indicated that supervision by the communities served performed better at 7.15 times as compared to those by medical trained personnel (95% CI = 2.832, 18.055). This is mainly because the community understands their own needs better; therefore, it is easier to give feedback and suggestions directly to the volunteers. As suggested by Meyer-Capps et al. [38], supervision which has traditionally been provided by the medical personnel, is too infrequently implemented because of their priority in patient-care and other administrative issues.

It is important to note the limitations of this study. This study used interview administered questionnaire in data collection; therefore response bias is unavoidable. In addition, it is a cross-sectional study and the findings do not establish the causal relationship between variables. As this study was carried out among the CHVs of Kuching district, the generalization of the result can only be done in another sample with similar background.

## 5. Conclusion

Nevertheless, despite the limitations, this study provides a preliminary finding on the assessment of role performance among CHVs of KOSPEN that will help the government to review and improve their strategies in community health. Factors such as age group, education level, marital status, prestige, and respect, seen as “doctor in community,” job aids, training location, and supervision by community members were some of the significant factors that can be further explored in the implementation of KOSPEN. To increase and maintain the current work force of CHVs as well the good performance requires commitment from all including the government, policy makers, stakeholders, and the communities. In line with the Alma Atta declaration of Primary Health Care Concept (PHC), all community-based health strategies must address the needs of the community, led by the community members themselves and for the communities.

## 6. Recommendation

As more countries look to scale up CHV programmes or shift additional tasks to CHVs, it is critical to pay attention to the elements that affect CHV productivity in the design phase as well as throughout implementation of a programme. An enabling work environment is crucial to maximize the productivity of CHVs. Policy makers, programme managers, and other stakeholders need to carefully consider how the productivity elements of workload, supportive supervision, availability of supplies and equipment, and respect are defined and incorporated in the overall CHV strategy.

Based on the finding, there were not many young generation of CHV, while majority of them belonged to the middle to elder groups. If this condition continue to happen, it will lead to shortage of CHVs' manpower in the future. Therefore, the concept of health volunteers should be expanded and implemented especially in younger age groups. A minimum of age requirement needs to be set in selection of the CHV. Besides that, from the result, it was shown that education background plays an important role in affecting their performances. The higher education background will have better understanding and knowledge, hence higher performances. Policy makers or programme manager should decide the minimal requirement for education level.

This study also highlighted that training is also an important factor in affecting the CHVs' services. Training should be competence and practice-based and located close to CHVs' working context. Training materials and activities should be specifically developed for CHVs rather than using training packages developed for facility-based workers. Besides, continuing or refresher training is as important as initial training. If regular refresher training is not available, acquired skills and knowledge are quickly lost, and that will influence their performance.

Based on the results, it was shown that supervision is often neglected in a health voluntary programme. Therefore, clear strategies and procedures are needed for supervision. There is a need to define what is the job scope and skills needed to be taught so that health personnel, CHVs and community health committee members know what is expected when they are selected as supervisor. The guidelines for supervision should include a list of supervisory activities. The most important element of supervision is ensuring the two-way flow of information between the supervisor and the health volunteers.

Provision of adequate job aids, equipment, health diaries, and others has been identified as crucial to CHV effectiveness. For health volunteers to be retained, managers of volunteer programme at district level should provide volunteers with appropriate means of transport and job aids for their work. This will help improve their performance as health volunteers. Nonmonetary incentives such as raincoats, torch lights, and boots should be provided by the district in-charges to the health volunteers. This will motivate volunteers to be more committed in their role of providing health care services to the door steps of their own community members.

## Figures and Tables

**Table 1:** Sociodemographic characteristic of volunteers (*n* = 210).

Sociodemographic variables	*n* (%)
Age group (years)	
24 and lower	14 (6.7)
25–34	44 (21.0)
35–44	65 (31.0)
45 and above	87 (41.4)
Mean = 42.61, min = 15, max = 71, and SD =12.85	

Gender	
Male	94 (44.8)
Female	116 (55.2)
Education level (completed up to)	
Primary	25 (11.9)
Secondary	152 (72.4)
Tertiary	33 (15.7)
Marital status	
Single	38 (18.1)
Married	172 (81.9)
Job occupation	
Full-timed salaried health volunteer	47 (22.4)
Business	15 (7.1)
Housewife	66 (31.4)
Civil servant	47 (22.4)
Full-timed farmer	35 (16.7)
Duration as health volunteers	
Less than 3 months	10 (4.8)
3–6 months	154 (73.3)
More than 6 months	46 (21.9)
Mean = 5.83, Min = 1, and max = 14	

**Table 2:** Motivation and attrition factor, knowledge, and organization input among community health volunteers (*n* = 210).

Variables	*n* (%)
*Motivating factors (yes)*	
To improve the health of others	204 (97.1)
Help members/sick people	203 (96.7)
To gain knowledge and practice skills learned	189 (90.0)
Proud with the programme	184 (87.6)
To make own community better	184 (87.6)
Incentives	149 (71.0)
Paid salary	80 (38.1)
Prestige and respect	79 (37.6)
Job opportunity from government	43 (20.5)
Seen as “doctor” in community	17 (8.1)
*Attrition/quit (yes)*	
No time	152 (72.4)
Transportation issue	137 (65.2)
Job aids (boots, raincoats, and medical equipment)	114 (54.3)
No support by community member	83 (39.5)
No respect by community member	59 (28.1)
*Knowledge (correct answer)*	
Smoking cigarettes is harmful to health	208 (99.0)
You will not be harmed if somebody smokes near you	169 (80.5)
Exercise will help you losing weight	209 (99.5)
Regular physical exercise causes you suffering from stroke, DM, hypertension	123 (58.6)
Healthy diet cannot control your weight	166 (79.0)
Healthy eating can cause cancer, DM, obesity	196 (93.3)
Obesity can be determined using BMI	195 (92.9)
Being overweight or obese do not harm our health	193 (91.9)
Exercise 30 minutes and quit smoking can reduce hypertension	198 (94.3)
Diabetes can lead to blindness	156 (74.3)
*Organizational input*	
Selection	
Ethnic leader	56 (26.7)
Village head	89 (42.4)
Community	50 (23.8)
Relative and family	8 (3.8)
Medical personnel	7 (3.3)
Duration of training	
Less than 1 day	21 (10.0)
1-2 days	166 (79.0)
More than 2 days	23 (11.0)
Adequacy of training	
Yes	148 (70.5)
Training location	
Community setting	46 (21.9)
Government setting	164 (78.1)
Supervision frequency	
Once a week	20 (9.5)
Once every 2 weeks	42 (20.0)
Once a month	148 (70.5)
Supervision by	
Community	141 (67.1)
Medical	69 (32.9)
Effectiveness of training	
Effective	52 (24.8)
Acceptable	112 (53.3)
Not effective	46 (21.9)
Supervision motivates me to work harder	
Yes	176 (83.8)
Supervision makes me more committed to the programme	
Yes	180 (85.7)

**Table 3:** Number and percentage of volunteers classified by role performance (*n* = 210).

Performance of CHV	*n* (%)
Above median	124 (59)
Below median	86 (41)
Median = 29, min = 9, max = 35, Q1 = 27, and Q3 = 32	

**Table 4:** Factors affecting role performance of health volunteer (*n* = 210).

Variables	Performance		Crude OR	Adjusted OR	95% CI	*p* value^*∗*^
	High^a^ *n* (%)	Low^b^ *n* (%)				
Age group (years)						**0.003**
24 and lower	7 (50.0)	7 (50.0)	18.52	27.29	2.781–267.945	**0.002**
25–34	26 (59.1)	18 (40.9)	2.35	3.50	1.127–10.878	0.078
35–44	47 (72.3)	18 (27.7)	3.20	4.93	1.784–13.660	**0.007**
45 and above (ref)	44 (50.6)	43 (49.4)	1.00			
Education level						<**0.001**
Primary (ref)	16 (64.0)	9 (36.0)	1.00			
Secondary	82 (53.9)	70 (46.1)	0.41	0.20	0.059–0.679	0.053
Tertiary	26 (78.8)	7 (21.2)	6.54	4.29	1.038–17.751	**0.044**
Marital status						
Single (ref)	18 (47.4)	106 (61.6)	1.00			
Married	106 (61.6)	66 (38.4)	4.08	4.65	1.006–17.588	**0.025**
Prestige and respect						
Yes	54 (68.4)	25 (31.6)	3.24	2.76	1.244–6.120	**0.012**
No (ref)	70 (53.4)	61 (46.6)	1.00			
Seen as “doctor” in community						
Yes	15 (88.2)	2 (11.8)	5.31	8.64	2.044–36.577	**0.003**
No (ref)	109 (56.5)	84 (43.5)	1.00			
Job aids (boots, raincoats, and medical equipment)						
Yes	72 (63.2)	42 (36.8)	1.75	3.04	1.324–7.008	**0.009**
No (ref)	52 (54.2)	44 (45.8)	1.00			
Training location						
Community setting	26 (56.5)	20 (43.5)	4.03	4.75	1.930–11.725	**0.001**
Government setting (ref)	69 (42.1)	95 (57.9)	1.00			
Supervision by						
Community	70 (49.6)	71 (50.4)	4.18	7.15	2.832–18.055	<**0.001**
Medical (ref)	25 (36.2)	44 (63.8)	1.00			

Hosmer and Lemeshow test *p* value = 0.088 > 0.05; CI = confidence interval; OR = odds ratio.

^*∗*^Multiple Logistic Regression (no multicollinearity, assumptions were all met)

Dependent variable = role performance (high performance versus low performance of CHV)

^a^High = above median of 29; ^b^Low = below median of 29.
